# Characteristics of primary care office visits to nurse practitioners, physician assistants and physicians in United States Veterans Health Administration facilities, 2005 to 2010: a retrospective cross-sectional analysis

**DOI:** 10.1186/1478-4491-10-42

**Published:** 2012-11-13

**Authors:** Perri A Morgan, David H Abbott, Rebecca B McNeil, Deborah A Fisher

**Affiliations:** 1 Department of Community and Family Medicine, Duke University Medical Center, Durham, NC, USA; 2 Health Service Research and Development Center of Excellence, Durham Veterans Affairs Medical Center, Durham, NC, USA; 3 Epidemiologic Research and Information Center, Durham Veterans Affairs Medical Center, Durham, NC, USA; 4 Department of Medicine, Duke University Medical Center, Durham, NC, USA

**Keywords:** Health manpower, Nurse practitioners, Physician assistants, Primary health care, United States Department of Veterans Affairs

## Abstract

**Background:**

Primary care, an essential determinant of health system equity, efficiency, and effectiveness, is threatened by inadequate supply and distribution of the provider workforce. The Veterans Health Administration (VHA) has been a frontrunner in the use of nurse practitioners (NPs) and physician assistants (PAs). Evaluation of the roles and impact of NPs and PAs in the VHA is critical to ensuring optimal care for veterans and may inform best practices for use of PAs and NPs in other settings around the world. The purpose of this study was to characterize the use of NPs and PAs in VHA primary care and to examine whether their patients and patient care activities were, on average, less medically complex than those of physicians.

**Methods:**

This is a retrospective cross-sectional analysis of administrative data from VHA primary care encounters between 2005 and 2010. Patient and patient encounter characteristics were compared across provider types (PA, NP, and physician).

**Results:**

NPs and PAs attend about 30% of all VHA primary care encounters. NPs, PAs, and physicians fill similar roles in VHA primary care, but patients of PAs and NPs are slightly less complex than those of physicians, and PAs attend a higher proportion of visits for the purpose of determining eligibility for benefits.

**Conclusions:**

This study demonstrates that a highly successful nationwide primary care system relies on NPs and PAs to provide over one quarter of primary care visits, and that these visits are similar to those of physicians with regard to patient and encounter characteristics. These findings can inform health workforce solutions to physician shortages in the USA and around the world. Future research should compare the quality and costs associated with various combinations of providers and allocations of patient care work, and should elucidate the approaches that maximize quality and efficiency.

## Background

Primary care, an essential determinant of health system equity, efficiency, and effectiveness [1], is threatened by inadequate supply and distribution of the provider workforce [2,3]. As the US primary care system confronts provider shortfalls due to demographic trends, the growing prevalence of chronic disease [4], and low proportions of physicians choosing primary care practice [5], a possible solution is expanded use of physician assistants (PAs) and nurse practitioners (NPs) [6]. This solution is supported by a large body of research demonstrating high quality of NP and PA care [7,8] and by recent research suggesting that higher proportions of NPs in primary care clinics are associated with improved outcomes among patients with diabetes [9,10].

The Veterans Health Administration (VHA), the United States’ largest integrated health system, is a leader in primary care innovation. Since the mid-1990s, the VHA has created a model primary care system by implementing strategies to coordinate and integrate care, maintain high standards of preventive and chronic disease care, make primary care accessible to veterans across the country, and provide high quality care while controlling costs [11-14].

Throughout this transformation, the VHA has explicitly promoted the use of NPs and PAs in primary care. The VHA is the largest employer of both PAs and NPs nationally [15,16]. Although deployment of NPs and PAs varies across regional VHA networks (Veterans Integrated Service Networks, or VISNs), many of these networks have been frontrunners in the utilization of nonphysician providers with respect to both numbers of PAs and NPs and to relative autonomy and responsibility in clinical care [15-17]. For example, VHA primary care NPs and PAs are typically responsible for management of their own panels of patients and are generally not required to obtain physician co-signatures for prescriptions, orders, or documentation [18,19]. Evaluation of the roles and impact of NPs and PAs in the VHA is critical in ensuring optimal care for veterans and may inform best practices for use of PAs and NPs in other settings. The VHA is a promising and pertinent system to study because of its unparalleled national system of coded data, the high burden of chronic disease in its patient population, and its relatively expansive use of PAs and NPs. The purpose of this study was to characterize the use of NPs and PAs in VHA primary care and to examine whether their patients and patient care activities were, on average, less medically complex than those of physicians.

## Methods

This is a retrospective cross-sectional analysis of national administrative data from VHA primary care encounters (2005 to 2010) listing a physician, NP, or PA as the first provider for the encounter. Other provider types (such as registered nurses, licensed practical nurses, pharmacists, and social workers) were the first provider listed for about 28% of all encounters and were omitted from the analysis. Encounters with physician residents were also excluded, but the number of visits for which a physician resident was listed as the first provider was small (less than 3% of total for all types). After we eliminated all provider types other than physicians, PAs, and NPs, the vast majority (>98%) of encounters in the dataset listed only one provider as involved in the encounter. Therefore, we analysed data for only the first provider listed. Our analysis of trends in the proportion of primary care visits attended by each provider type from 2005 to 2010 is based on 9.6 million to 10.6 million encounters from each year. For all of the other analyses, we used only 2010 data, comprising 10.6 million encounters.

Variables analysed by provider type included patient age, sex, race, VISN, visit primary diagnosis by International Classification of Diseases (ICD-9) code, procedures by Current Procedural Terminology (CPT^®^) code, and comorbidity score. Encounter primary diagnoses (ICD-9 codes) were aggregated into 288 categories using the Health Cost and Utilization Project Clinical Classification Software [20], and then further categorized into 30 clinical categories by our team. The comorbidity score system used was that of the diagnostic cost groups (DCG), which standardizes risk compared with the average Medicare patient (DCG score = 1), where a score >1 indicates that the patient studied has a higher health risk than the average Medicare patient. This score was pre-calculated for each patient by VHA health services researchers and was obtained through the VHA Information Resource Center (VIReC).

Statistical analysis was descriptive and accomplished using SAS Version 9.2 (SAS Institute, Cary, NC). The extremely large size of our dataset produced highly precise estimates, even for differences of trivial magnitude and no clinical consequence. For this reason, and because our approach to the analysis was descriptive (rather than modelling), we chose to present summary statistics without confidence intervals or estimates of statistical significance.

This study was approved by the Durham Veterans Affairs Medical Center Institutional Review Board, which found that it complied with ethical and regulatory standards.

## Results and discussion

### Trends and numbers of patient encounters by provider type

A substantial portion (29%) of VHA primary care encounters are with PAs and NPs. Nurse practitioners are more prominent than PAs in VHA primary care, attending approximately twice as many visits as PAs (19.2% versus 8.4% in 2010). This mirrors the non-VHA distribution, since larger numbers of NPs than PAs practice in primary care [21]. Our study cannot determine whether the predominance of NPs over PAs is due to supply factors, such as possible PA preference for subspecialty practice, or to demand factors, such as preferential recruitment of NPs for primary care positions.

**Figure 1:**
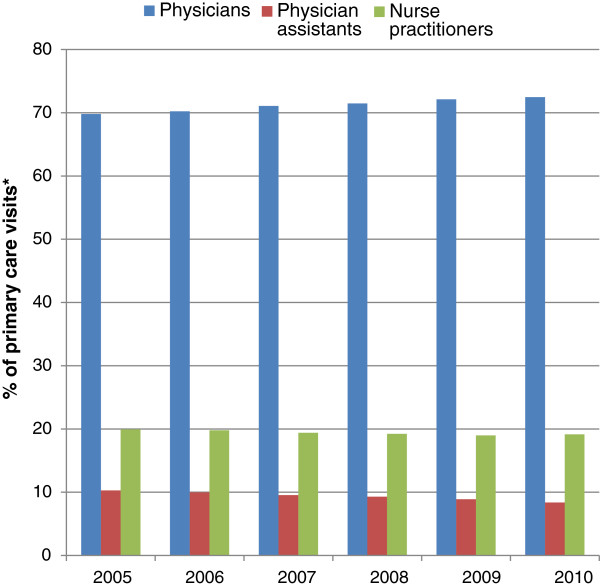
**Trend in percentage of primary care visits by provider type, 2005 to 2010.** *Only visits to physicians, nurse practitioners, and physician assistants were included.

The annual number of VHA primary care encounters involving the three provider types increased from 9.6 million to 10.6 million between 2005 and 2010. Almost all of this increased workload was absorbed by physicians, whose annual primary care encounters increased from 6.7 to 7.7 million annually. The percentage of total encounters attended by physicians increased from 69.8% to 72.5% over the six years studied, with corresponding minor decreases in the percentages seen by NPs and PAs (Figure 1).

### Regional variation in use of NPs and PAs

**Figure 2:**
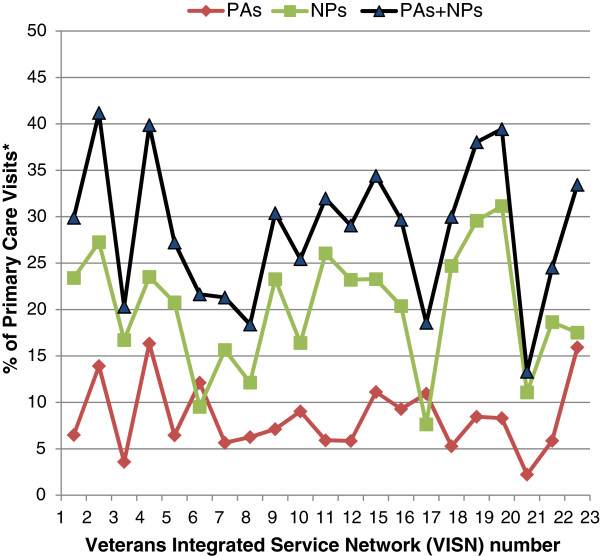
**Variation in number of VHA primary care visits to PAs and NPs by regional network (VISN), 2010.** *Only visits to physicians, nurse practitioners, and physician assistants were included.

The use of NPs and PAs varies widely by regional network (VISN), with the two provider types together attending as few as 13% (VISN 21) and as many as 41% (VISN 2) of primary care encounters in 2010 (Figure 2). In some regional networks, such as VISN 2, both NPs and PAs see relatively large numbers of patients (27% of encounters for NPs and 14% for PAs). In most VISNs, NPs attend substantially more encounters than do PAs, up to about four times as many in VISN 20 (31% for NPs and 8% for PAs). However, in two VISNs (VISN 6 and 17), PAs attend slightly more visits than NPs (12% and 11% for PAs versus 10% and 8% for NPs respectively). We did not examine variability at the facility level, which may also be extensive. This variability may provide an opportunity for comparative research across a spectrum of PA and NP use.

### Patient and encounter characteristics

In 2010, the distribution of patient age, sex, and race was fairly constant across the provider groups (Table 1). The mean age of patients whose visits were attended by physicians (62.8 years) was minimally higher than that of patients seen by NPs (61.7years) or PAs (61.1 years). Nurse practitioners saw slightly more women (10% of patient encounters) than did PAs (6.7%) and physicians (6.6%). Slightly more visits to physicians and NPs were by patients from minority groups (21 and 20%, respectively) compared with visits to PAs (18%). Differences in proportions of encounters with patients of racial and ethnic minorities may be due to geographical differences in PA and NP use. The purpose of the visit varies by provider type, with PAs seeing more patients for physical examinations to determine eligibility for benefits (9%) than physicians (3.4%) or NPs (5.2%). Physician assistants also saw more unscheduled patients (5.3%) than did physicians (4.2) or nurses (4.5%).

**Table 1:** Comparison of 2010 VHA primary care encounter^**a **^characteristics by provider type

	**Physician**	**Physician assistant**	**Nurse practitioner**
Mean age (years)	63	61	62
Male (%)	93	93	90
Race or ethnicity (%)			
Non-Hispanic white	58	61	58
Non-Hispanic black	14	13	14
Hispanic	6	3	4
Asian	1	1	1
American Indian	1	1	1
Unknown	21	21	22
Purpose of visit (%)			
Scheduled clinic visit	92	86	90
Unscheduled visit	4	5	5
Physical examination to determine compensation and pension eligibility	3	8	5
Current Procedural Terminology (CPT^®^) codes (%)			
Evaluation and management, established patient	76	71	72
Evaluation and management, new patient	5	6	5
Immunization	7	6	6
Disability evaluation	3	8	5
Lifestyle counselling	2	1	2
Preventive medicine	1	2	1
Missing and other	6	6	8
Mean diagnostic cost group scores^b^ (s.d.)	0.89 (0.891)	0.82 (0.824)	0.84 (0.821)

^a^ Only visits to physicians, nurse practitioners, and physician assistants were included.

^b^ DCG = diagnostic cost group, a comorbidity measure based on age, sex, and diagnoses over a one-year time period. Scores are standardized so that a value of one is the score of a typical Medicare patient.

Nurse practitioner and PA patients had slightly lower DCG complexity scores than physician patients (physicians, 0.89; NPs, 0.84; PAs, 0.82). The differences in the DCG scores are quite small compared with the standard deviation of these measures, suggesting that the scores can be considered similar across the three provider groups. All three groups saw patients with lower DCG scores than the average Medicare patient, probably because the VHA population includes many people in the under-65 age group. The finding of only small differences in this measure of patient complexity challenges the prevailing notion that NPs and PAs see patients who are less medically complex than those cared for by physicians. Since our study did not address referral rates by provider types, we cannot assess whether PAs or NPs were more likely to refer complex patients to specialists. Analysis of referral rates and appropriateness of referrals will be important in future evaluations addressing both quality of care and cost efficiency by provider type.

**Figure 3:**
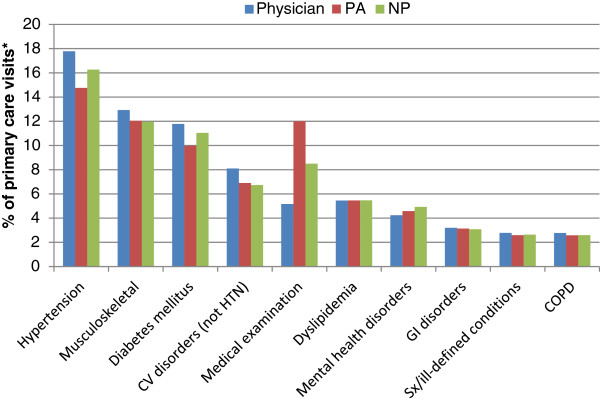
**Percentage of visits to each provider type by primary visit diagnosis (10 leading diagnoses by frequency), 2010.** *Only visits to physicians, nurse practitioners, and physician assistants were included. Cardiovascular disorders exclude hypertension since hypertension is a separate category. COPD = chronic obstructive lung disease.

The most commonly seen 2010 primary visit diagnoses were similar across provider groups (Figure 3). The two leading diagnoses for all provider types were hypertension and musculoskeletal conditions. For physicians and NPs, the third most common diagnosis was diabetes mellitus, but for PAs the third most common diagnosis was “general medical examination”, followed by diabetes mellitus. Physician assistants had notably more visits in the category of “medical examination” (12% of all visits to PAs) than NPs (8.5%) and physicians (5.2%). For all other diagnoses, the proportion of each provider type’s visits agreed within 2% (absolute).

**Figure 4:**
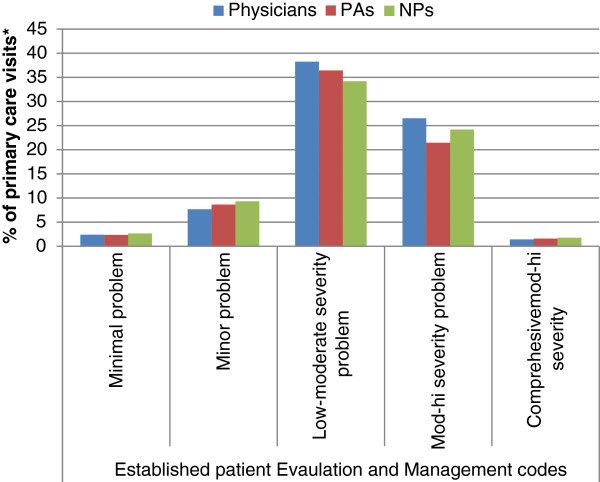
**Evaluation and management, established patient Current Procedural Terminology (CPT^®^) codes: proportions by provider type, 2010.** *Only visits to physicians, nurse practitioners, and physician assistants were included. Bars represent the percentage of visits to each provider type that are within the indicated CPT^®^ code.

**Figure 5:**
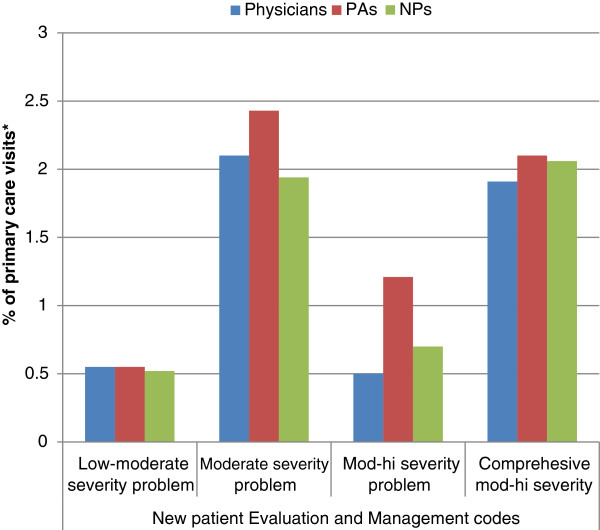
**Evaluation and management, new patient Current Procedural Terminology (CPT^®^) codes: proportions by provider type,2010.** *Only visits to physicians, nurse practitioners, and physician assistants were included. Bars represent the percentage of visits to each provider type that are within the indicated CPT^®^ code.

Procedure codes for patient visits were heavily concentrated in the evaluation and management (E/M) categories, particularly for established patients. Physician assistants performed more disability evaluations and saw more new, as opposed to established, patients for E/M encounters than did physicians. In addition, PAs had correspondingly fewer encounters with established patients than did physicians or NPs. Nurse practitioners fell between physicians and PAs on numbers of encounters in these three categories (established patients, disability evaluations, and new patients). Within encounters for established patients, physicians staffed slightly more visits towards the more complex end of the spectrum than did NPs or PAs (Figure 4). For new patients, PAs attended higher proportions of the most complex encounters (Figure 5).

Overall, NPs, PAs, and physicians filled similar roles in VHA primary care clinics, although there were some differences in patient complexity and purpose of visits. The similarities in the patterns of patient encounter characteristics across provider types suggests that NPs and PAs function more as physician substitutes than as physician complements [8] in VHA primary care. Both provider types, however, have found specific patient care niches. Although the proportion of women patients in the VHA remains small, NPs attended more visits with these female patients. The finding that PAs attended more unscheduled visits suggests that PAs may often be used to staff walk-in or same-day appointment sections of primary care clinics. This deployment of PAs could also explain why they saw proportionately more new patients with higher complexity, since ill veterans who present to obtain care for the first time may be routed through sectors of the practice set aside for unscheduled appointments.

Physician assistants and, to a lesser extent, NPs also saw more visits for the purpose of determining benefit eligibility than did physicians. While these eligibility visits are detailed and are important to veterans’ financial futures, they are routine in nature and generally do not address emergent conditions. Therefore, assigning these visits to less expensive and less highly trained providers may be an efficient use of human resources.

### Study strengths and limitations

Our results are strengthened by the high quality of the medical record data we used. The data are national in scope, reflecting the experience of veterans across the country. Data were recorded as part of routine administrative processes at or near the time of patient encounters, removing recall as a source of bias. Perhaps most importantly, PA and NP providers within VHA directly document their own patient encounters, so our analysis did not suffer from the common practices, such as billing “incident to” the physician, which can obscure PA and NP patient care activities in administrative datasets.

It is possible that PAs and NPs saw patients jointly with physicians more than the data reflect. The scarcity (<2%) of encounters that coded multiple providers of interest (physician, PA, NP) may be an artefact of routine practices in which the documenting provider does not code other providers who may have seen the patient. This practice may also explain why care by medical residents is not well-represented in the data. Given the substantial teaching mission of the VHA, physician resident participation may have been much larger than the 3% of visits for which a resident was listed as the primary provider.

The large regional differences that we found in the use of NPs and PAs in VHA primary care could influence our results. As we discussed, this regional variation in NP and PA use probably affects the race and ethnicity differences that we found in the proportions of patients seen by each provider type. These regional variations could mask differences that are not apparent in our analysis.

The generalizability of our results is influenced by a number of factors. Most VHA providers are salaried, and may therefore behave differently than providers in the private sector, whose income may depend on patient and procedure volume. Moreover, VHA patients have a higher burden of chronic disease than the general US population. However, information about the use of NPs and PAs in caring for a population with a high prevalence of chronic disease can inform workforce planning for other similar settings. This is important for health workforce policy, since chronic disease accounts for over half of US health-care expenditure [22].

### Future research

While our study elucidates patient care activities of NPs and PAs in primary care in the VHA, future research should establish which allocations of labour maximize quality and efficiency. The large variation that we found in the magnitude of PA and NP use across regional VISN networks suggests that there may also be variation in the *pattern* of NP and PA use. This variation, while hidden within our nationally aggregated results, could present opportunities for research on the best use of PAs and NPs through comparison of use and outcomes across facilities or VISNs that use PAs and NPs differently. The VHA primary care data also support analysis at the team level, which was beyond the scope of this project but which could support important analyses of the effects of team structure and composition on outcomes.

## Conclusions

Primary care physician shortages currently exist or are expected around the world. In response, many nations are exploring or developing roles for nonphysician providers, and information about current primary care use of NPs and PAs is highly relevant to those endeavours.

Our study describes a large integrated health system that uses NPs and PAs to fill patient care roles similar to those of physicians. These results demonstrate that a highly successful nationwide primary care system relies on NPs and PAs to provide over one quarter of primary care visits to a patient population with a high prevalence of chronic disease. Future research should compare the quality and costs associated with various combinations of providers and allocations of patient care work, and should elucidate the approaches that maximize quality and efficiency.

## Abbreviations

COPD: Chronic Obstructive Lung Disease; CPT^®^: Current Procedural Terminology; DCG: Diagnostic Cost Group; E/M: Evaluation and management; ICD-9: International Classification of Diseases; NP: Nurse Practitioner; PA: Physician Assistant; VHA: Veterans Health Administration; VIReC: VHA Information Resource Center; VISNs: Veterans Integrated Service Networks.

## Competing interests

The authors declare that they have no competing interest.

## Authors’ contributions

PAM conceived the study, provided leadership for the study design, and drafted the manuscript. DHA performed the data analysis. All authors participated in design of the study, contributed to interpretation of results, provided substantive critique of manuscript drafts, and approved the final manuscript.
